# Visceral adipose tissue alteration of *PI3KR1* expression is associated with gestational diabetes but not promoter DNA methylation

**DOI:** 10.1080/21623945.2019.1675239

**Published:** 2019-10-12

**Authors:** Rebecca C. Rancourt, Raffael Ott, Karen Schellong, Kerstin Melchior, Thomas Ziska, Wolfgang Henrich, Andreas Plagemann

**Affiliations:** a Division of ‘Experimental Obstetrics’, Clinic of Obstetrics, Charité – Universitätsmedizin Berlin, Humboldt-Universität zu Berlin, and Berlin Institute of Health, Berlin, Germany; b Clinic of Obstetrics, Charité – Universitätsmedizin Berlin, Humboldt-Universität zu Berlin, and Berlin Institute of Health, Berlin, Germany

**Keywords:** Gestational diabetes mellitus, epigenetics, DNA methylation, phosphatidylinositol 3-kinase, adipose tissue

## Abstract

Obesity and diabetes are at an epidemic rate, as well as growing incidences of gestational diabetes mellitus (GDM) which causes pregnancy risks, and harm in both maternal and child health. It remains unclear which molecular mechanisms are driving the functional differences between visceral and subcutaneous fat and how these types directly affect an individual’s health outcome. Paired abdominal subcutaneous and omental visceral adipose tissue were collected from women with GDM (n = 20) and with normal glucose tolerance (NGT, n = 22) during planned caesarian section. Both groups had similar maternal age (average 32.5 years) and BMI at delivery (average 33.3 kg/m^2^). Adipose tissue mRNA expression analyses of insulin signalling genes: *PI3KCA, PI3KR1, IRS1 and IRS2* showed significantly decreased *PI3KR1* expression (−23%) in visceral fat in GDM with no association to promoter DNA methylation. Reduced visceral fat *PI3KR1* expression appears to be a pathogenic factor in GDM but not through altered promoter methylation.

## Introduction

Overweight and obesity are enormous public health problems leading to various deleterious downstream health conditions. Furthermore, this prevalence of overweight and obesity is growing in cases of pregnancy as well as the rising trend of gestational diabetes mellitus (GDM) which ultimately lead to increased risk for future health complications for mother and child [1–8]. The distribution/relative proportion of adipose tissue is a major determinant factor of an individual’s metabolic health. Adipose tissue functions to store energy, serves as important endocrine organ, and various studies have shown dysregulated fat linked to metabolic dysfunction [9,10]. Especially, visceral adipose tissue (VAT) has been linked to chronic diseases. It remains unclear which mechanisms and, possibly, epigenetic modifications are driving the functional differences between visceral and subcutaneous fat, as well as how these fat types directly influence health outcomes, *e.g*. gestational diabetes.

This study is a continuation of our previously reported research involving the gene expression and epigenetic regulation of the metabolic players, insulin receptor (*IR*) and adiponectin, in this biologically/functionally relevant tissue (maternal adipose) within pregnancy conditions such as GDM, overweight and obesity [11,12]. Here we investigate the possible relevance of key downstream signal pathway factors of *IR*, in particular: insulin receptor substrate 1, (*IRS1*); insulin receptor substrate 2, (*IRS2*); phosphoinositide-3-kinase subunit p85, (*PI3KR1*); and phosphoinositide-3-kinase alpha (*PI3KCA*). These transcription factors play important roles in the metabolic actions of insulin (*e.g*. glucose transport) and are essential for adipocyte differentiation. Of the insulin receptor substrate family, *IRS1* and *IRS2* are the two most ubiquitously expressed and can bind to the PI3K (especially the p85 subunit) signalling protein [13–15]. Both *PI3KR1* (regulatory subunit) and *PI3KCA* (catalytic subunit) need to come together to work as an enzyme[15]. Disruption in the PI3K-linked pathway has been identified in obesity as a contributor to insulin resistance[14]. We sought to examine the mRNA expression levels of the aforementioned genes in both subcutaneous and visceral adipose tissue from mothers with GDM compared to pregnancies with normal glucose tolerance (NGT). Any changes in expression were further investigated with analysis of promoter DNA methylation to determine whether this epigenetic modifier influenced/regulated the altered gene expression.

## Materials and methods

### Subject data

This research is part of the prospective observational ‘Early CHARITÉ (*EaCH)*’ cohort study [11,12,16]. Here, we investigated additional genes involved in the insulin pathway on just those cases in which the optimal material and factors (such as transcription, metabolic and hormonal) could be measured in a complete set in order to avoid missing data bias. This resulted in a subgroup of the previously reported studies on adiponectin and insulin receptor genes [11,12]. Twenty women with GDM and 22 women with NGT were prospectively recruited before scheduled Caesarean section (CS) of singletons at the Clinic of Obstetrics of the Charité – Universitätsmedizin Berlin, Campus Virchow-Klinikum, Germany. Recruitment, exclusion criteria, standardized procedures, analytical methods *etc*. are described in detail elsewhere [15]. Groups were matched for maternal age, ethnic origin, socio-economic status (SES), parity and, in particular, prepregnancy BMI. BMI was calculated with maternal height and weight before conception and the last measured weight within 1 week prior to delivery and then categorized according to the WHO criteria (normal weight: 18.5–24.9 kg/m2, overweight: 25.0–29.9 kg/m2, obese: ≥30.0 kg/m2). GDM screening was performed between the 24–28^th^ week of gestation according to national guidelines at the time of recruitment [17,18]. Within the GDM group, nine women were treated by diet and eleven were treated with diet and additional insulin therapy to achieve glycaemic control. No oral antidiabetic drugs were administered. Further clinical parameters such as plasma insulin, C-peptide, glucose and homeostatic model assessment of insulin resistance (HOMA-IR) were determined for the cases here as described elsewhere [11,12,16,19]. Research design and methods were conducted in accordance to the Declaration of Helsinki, revised in 2004, and approved by the local Ethics Committee (EA2/026/04). Informed written consent was obtained from all subjects.

### Adipose tissue sampling

Paired maternal sample biopsies of subcutaneous adipose tissue (SAT) from the abdominal anterior wall and VAT omental samples (from the greater omentum [20]) were obtained during CS delivery, snap frozen in liquid nitrogen and stored at −80°C. Samples of ~200 mg in size were available in total from all subjects investigated here.

### Gene expression analyses

Total RNA was isolated from adipose tissue (100 mg) using the RNeasy Lipid Tissue Mini Kit (Qiagen, Hilden, Germany) according to manufacturer’s protocol. Quantity and purity were assessed with a spectrophotometer (NanoDrop 1000, Thermo Scientific, Wilmington, DE, USA) and with a Bioanalyzer 2100 (Agilent Technologies, Santa Clara, CA, USA). Adipose tissue RNA (300 ng) was reverse transcribed with the iScript kit (Bio-Rad, Hercules, CA, USA). Quantitative real-time PCR was performed in triplicate using TaqMan technology (Applied Biosystems, Waltham, MA, USA) and a 7500 instrument (Applied Biosystems) included respective controls and quality checks. Pre-designed exon-exon spanning TaqMan primer assays from Applied Biosystems were used (ID: *IRS1*: Hs00178563_m1; *IRS2*: Hs00275843_s1; *PIK3R1*: Hs00933163_m1; *PIK3CA*: Hs00907957_m1) and amplified in singleplex with the housekeeping gene peptidylprolyl isomerase A (*PPIA*: Hs99999904_m1) [21]. Gene expression was normalized using the 2^−Δ*C*t^ method, including correction for amplification efficiency calculated from the standard curves for each primer set [22,23]. As in previous reports [11,12], gene expression of *PPIA* showed no group differences in both VAT and SAT. In addition to the individual gene mRNA expression levels, the net sum of *PI3KR1* + *PI3KCA* was included since the PI3K-‘complex’ together acts in distinct functional and molecular concert at the respective pathway downstream of *IR* [13,15]. The net sum of the insulin receptor substrate family members 1 and 2 (*IRS1* + *IRS2)* were also, respectively, included [15].

### DNA methylation analyses

Genomic DNA was extracted from 30 mg VAT, using the Genomic DNA-Tissue kit and the Quick-gDNA Blood kit (Zymo Research, Irvine, CA, USA), according to manufacturer’s protocols. Bisulphite conversions were performed using the EZ DNA Methylation Gold kit (Zymo Research). *In silico* analyses revealed a CpG island at the *PI3KR1* promoter region (Chromosomal location chr5:67,511,584–67,597,649; UCSC Genome browser on human Feb. 2009, GRCh37/hg19 assembly). The target region was selected as the only characterized transcription factor binding site (TFBS) PPARgamma lays within this sequence. Methylation assays were designed with PyroMark Assay Design Software v. 2.0 (Qiagen), primer and assay information is as follows (5ʹ to 3ʹ- Bisulphite converted sequence): Forward primer: TTGTTTTGTTATTTTAGGATTAGAAGTTAT, Reverse biotinylated primer: AAAACCAAAAAACCCTACAATTCCTAATCT, sequencing primer 1: AGGTTGTAGGAGTTAG and sequencing primer 2: GTTTATTTTTTTATTTTTTTTTTAT (amplicon size 256 base pairs and annealing temperature is 54°C). Pyrosequencing was performed on amplified PCR products using the PyroMark Q24 pyrosequencer (Qiagen). Percent methylation was analysed across individual measured CpG sites (12 sites). Assay reproducibility and specificity was validated using duplicate samples, various tissue types, and methylation scales (0–100%).

### Statistical analyses

Data are presented as means ± SEM or number and percentage. Group comparisons were analysed by unpaired *t*-test or Mann-Whitney-*U*-test as appropriate. Spearman’s correlation coefficients (r) were calculated to assess associations between clinical and/or endocrine parameters, gene expression and DNA methylation, respectively. Partial Pearson’s coefficients correlations (R) were used to check potential confounding effects of maternal BMI (prepregnancy and at delivery) and maternal age. Statistical analyses were performed with GraphPad Prism 7.00 (GraphPad Software, San Diego, CA, USA) and SPSS 23.0 software (IBM, Munich, Germany). Statistical significance was set at *P* < 0.05.

## Results

### Study cohort

Maternal and birth characteristics according to the study groups (NGT, n = 22 and GDM, n = 20) are reported in Table 1. Maternal age and BMI at both prepregnancy and at the time of delivery was similar between groups. Both groups were categorized as overweight according to the mean prepregnancy BMI (NGT, 26.8 and GDM, 28.4). For the GDM group, maternal metabolic and hormonal state remained altered (*e.g*. hyperinsulinemia and hyperglycaemia) at the end of pregnancy as compared to controls (*e.g*. C-peptide, insulin and glucose plasma levels). According to HOMA-IR, women with GDM exhibited higher insulin resistance compared to the NGT group (GDM, 8.2 *vs*. NGT, 3.2, *P* = 0.004).

**Table 1.** Maternal and birth characteristics of study cohort according to groups (NGT and GDM).

	NGT	GDM	
*n*	22	20	*P*-value*
Maternal age (years)	32.0 ± 1.1	33.0 ± 1.0	0.55
Prepregnancy BMI (kg/m^2^)	26.8 ± 1.7	28.4 ± 1.5	0.20
BMI at delivery (kg/m^2^)	33.1 ± 1.9	33.5 ± 1.5	0.40
Blood glucose at oGTT (mg/dL)			
Fasting	79.5 ± 1.7	100 ± 6.7	**<0.0002**
1-h	121 ± 6.3	213 ± 8.0	**<0.0001**
2-h	90.3 ± 4.2	166 ± 10.8	**<0.0001**
Area under the curve (mg/dL*h)	206 ± 8.2	346 ± 15.6	**<0.0001**
Maternal fasting plasma levels at delivery:			
Glucose (mg/dL)	71.1 ± 2.3	85.0 ± 1.2	**<0.0001**
Insulin (µU/mL)	21.5 ± 3.4	40 ± 8.0	**0.04**
HOMA-IR	3.2 ± 0.3	8.2 ± 1.6	**0.004**
C-peptide (ng/mL)	2.0 ± 0.2	4.8 ± 0.6	**<0.0001**
Infant data:			
Birth weight (g)	3365 ± 106.0	3581 ± 98.9	0.059
Relative Birth weight (g/cm)	66.4 ± 1.5	70.31 ± 1.7	0.075

Data are means ± SEM.

Abbreviations: NGT: Normal glucose tolerance, GDM: gestational diabetes mellitus, BMI: Body-mass-index, oGTT: Oral glucose tolerance test, HOMA-IR: Homoeostatic model assessment of insulin resistance

*Statistical significant (*P*-value<0.05).

### Targeted gene expression analysis of downstream insulin signalling pathway genes

**Figure 1.**
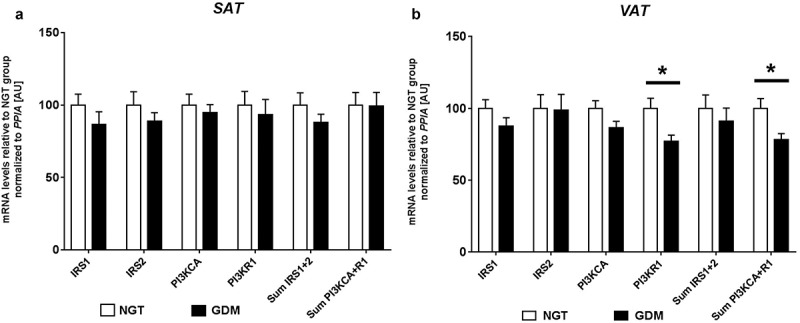
Relative mRNA levels of *IRS1, IRS2, PI3KCA* and *PI3KR1* in SAT (a) and VAT (b) of women with GDM *vs*. NGT. Relative gene expression of insulin receptor substrate 1 and 2 (*IRS1, IRS2)*, phosphoinositide-3-kinase alpha (*PI3KCA*) and phosphoinositide-3-kinase regulatory subunit p85 (*PI3KR1*) was normalized to peptidylprolyl isomerase A *(PPIA)* in abdominal subcutaneous (SAT- a) and visceral omental adipose tissues (VAT – b), of women with gestational diabetes mellitus (GDM; black; n = 20) *vs*. normal glucose tolerant women (NGT; white; n = 22). Sum expression values of *IRS1 + 2* and *PI3KCA+R1* were also included. Data are means ± SEM, shown as percentage to NGT levels. A.U., arbitrary units.* *P*< 0.05 (Mann-Whitney-U test). *PI3KR1-VAT P* = 0.012, Sum *PI3KCA+R1*-VAT *P* = 0.014.

Relative mRNA expression analyses were performed in both SAT and VAT for *IRS1, IRS2, PI3KR1*, and *PI3KCA*. In both fat types, no significant differences between groups were observed for *IRS1, IRS2* and *PI3KCA* (Figure 1). Overall, relative expression values were similar between VAT and SAT for *IRS1* and *PI3KCA*. In VAT, mRNA gene expression of *PI3KR1* (p85) was significantly reduced in women with GDM (GDM: 1192 ± 49 *vs*. NGT: 1543.6 ± 110, *P* = 0.012). No difference in *PI3KR1* was observed in SAT (Figure 1). When comparing the net-sum expression values of *PI3KCA+R1* in VAT, a significant difference was found (GDM: 1371 ± 56 *vs*. NGT: 1751 ± 119, *P* = 0.014). These differences resulted in −23% *PI3KR1* and −22% *PI3KCA+R1* less mRNA expression in diabetic subjects as compared to controls in VAT. In general, *PI3KR1* was more highly expressed in VAT compared to SAT.

### *DNA methylation at the* PI3KR1 *promoter in visceral adipose tissue*

**Figure 2.**
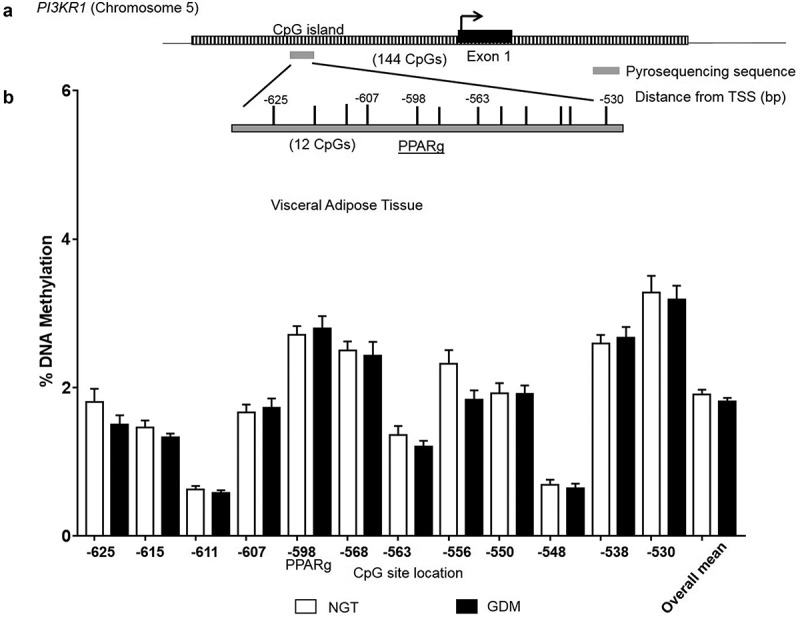
DNA Methylation analysis in the *PI3KR1* promoter region. CpG site-specific DNA methylation analyses at the phosphoinositide-3-kinase regulatory subunit p85 (*PI3KR1*) at the CpG island within the promoter region in the visceral omental adipose tissue from mothers with NGT vs. GDM. Schematic illustration of the DNA methylation assay for the *PI3KR1* promoter region, including a characterized transcription factor binding site for PPARgamma (a). Percent DNA methylation at each individual CpG site investigated (12 CpG sites) in the VAT for normal glucose tolerant (NGT; white; n = 22) *vs*. gestational diabetes mellitus group (GDM; black; n = 20). (b) CpG site number is location distance from transcriptional start site (TSS). Overall mean across CpG sites is also included. Data are means ± SEM.

DNA methylation analysis was performed in VAT to investigate a possible epigenetic-mechanistic change (according to groups) in the promoter methylation which could alter gene expression. The overall DNA methylation pattern at *PI3KR1* promoter was similar across VAT samples with all 12 CpG sites investigated showing low methylation levels (<5%) (Figure 2). No significant differences were found between groups at individual CpG sites or with the overall mean (GDM: 1.8% *vs*. NGT: 1.9%; Figure 2). Additionally, mRNA VAT expression levels did not significantly correlate with corresponding methylation levels at the individual CpG sites or the overall mean (CpG overall mean: Spearman r = 0.21, *P* = 0.17).

## Discussion

Our data further support that differences at a molecular level occur in visceral *versus* subcutaneous adipose tissue in the context of GDM. This is first reported analysis of *PI3KR1* mRNA expression in maternal VAT. Disruption of *PI3KR1* expression in VAT is most likely influenced by the mother’s insulin resistance and/or *vice versa*. This difference in expression, further builds to our previous research showing altered gene expression of insulin receptor and adiponectin in maternal VAT according to GDM and speaks to the importance of using biologically/functionally relevant adipose tissue in building a complete maternal genomic profile for target genes in GDM [11,12].

While *PI3KR1* and the sum of *PI3KR1* plus *PI3KCA* were reduced in VAT of GDM patients, we found no differences in abdominal SAT between GDM and NGT groups in the insulin pathway players we targeted (*IRS1, IRS2, PI3KR1 and PI3KCA*). This is a similar finding to a previous study looking at mRNA expression in SAT of women with and without polycystic ovary syndrome (a condition also characterized by increased insulin resistance) in which no differences in these genes were observed according to groups [24]. Again speaking to the differing roles/profiles the fat types have. Accordingly, it should be noted that our findings apply to abdominal subcutaneous fat and may not be representative of other body fat deposits such as buttocks, *etc*. The altered mRNA levels between experimental groups may be related to fatty acid profiles in visceral adipose tissue as discussed by Petrus *et al.* [25]. Understanding how adipose tissue (especially visceral) acts as a diseased organ should enable more strategic development for prediction, prevention and possible treatment measures, especially concerning GDM.

The PI3K pathway is essential for various cellular functions (*e.g*. apoptosis, cell growth, etc.) and has been well studied for its roles in cancer biology (*e.g*. endometrial cancer), especially the potential target drug/inhibitors for therapeutic approaches [15,26,27]. However, more research has shifted focus to the PI3K pathway’s role in adipocyte differentiation, inflammation and insulin sensitivity [13]. There is strong association between metabolic health/complications and increased risks of chronic disease (*e.g*. hypertension, stroke, etc.). There are several overlapping pathophysiological processes/factors that *PI3KR1* influences across these health conditions which make understanding how molecular mechanisms act (*e.g*. epigenetic modifications) all the more important. Despite *PI3KR1* importance in metabolic health, it is surprising that expression and DNA methylation in VAT has not been previously reported (Table 2). A summary of the current molecular findings for *PI3KR1* in studies involving metabolic health (*e.g*. GDM) is provided in Table 2.

**Table 2.** Summary of PI3KR1 (p85) molecular findings in studies involving metabolic health *e.g*. GDM and adiposity.

Author and Year ^(Reference)^	Model system/Study Design	Sample size	Samples-Tissue	Method: Molecular analysis/assay	Findings/Main results
Catalano *et al*. 2002^[14]^	Human	GDM; n = 5, pregnant-Control; n = 4	Abdominal SAT	Expression: Protein (Western-blot)	PI3Kp85α protein higher in GDM
Colomiere *et al* 2009^[31]^	Human, Clinical Study	GDM insulin- or diet-controlled, Control; n = 6–7 per group	Placenta	Expression: mRNA (RT-PCR), Protein (Western-blot)	Decreased mRNA and protein expression in GDM insulin-controlled group
Chu *et al*. 2010^[30]^	Human, cohort	GDM, Control; n = 50 per group	Blood, Adipose tissue (type not specified)	Expression: mRNA (RT-PCR), Protein (Western-blot)	No differences
				PI3K activity (immunoprecipitation)	Decreased activity in GDM
Veenendall *et al*. 2012^[33]^	Human, cohort	Prenatal famine exposure, unexposed n = 757	Blood	DNA Methylation (Methylation sensitive PCR)	No differences
Zhang *et al*. 2014^[34]^	Human, cohort	GDM, Control; n = 45 per group	Skeletal muscle	Expression: Protein (Immunohistological staining)	Protein higher in GDM
				PI3K activity (ELISA)	Decreased activity in GDM
Xu *et al*. 2015^[24]^	Human, cohort	PCOS; n = 22, Control; n = 13	Abdominal SAT	Expression: mRNA (quantitative RT-PCR)	No differences
Zhang *et al*. 2016^[32]^	Human, cohort	GDM, Control; n = 45 per group	Placenta	Expression: mRNA (RT-PCR), Protein (Western-blot)	No differences

Abbreviations: GDM: gestational diabetes mellitus, SAT: Subcutaneous adipose tissue, PCOS: Polycystic ovary syndrome

Additionally, we therefore provide for the first time, a DNA methylation profile across CpG sites within the *PI3KR1* promoter in VAT in the context of pregnancy, obesity and GDM. Interestingly, no difference in DNA methylation or correlation to mRNA was detected across the promoter region investigated here. Within the target DNA methylation assay is the putative peroxisome proliferator response element (PPRE) 2 which was previously identified in cell culture (*in vitro*) experiments with 3T3-L1 preadipocytes and was shown to play a role in transcriptional activation of *PI3KR1 via* the transcription factor binding of peroxisome proliferator-activated receptor gamma (PPARgamma)[28]. Overall transcriptional regulation is not well characterized for *PI3KR1*, except for the PPARgamma site, so DNA methylation emerged as putative regulatory factor. However, as the PPRE2 sequence does not contain any CpG sites and as methylation is not altered, this lends to DNA methylation not having a direct regulatory influence. We propose that perhaps modifications at the next regulatory-level at the chromatin (*e.g*. histone modifiers) across the PPRE sequences could be factors in potentially opening or closing transcription activating binding or enhancer elements in a tissue-specific manner. Another possible mechanistic candidate is microRNAs (miRNAs), which can play a role in driving tissue-specific expression patterns. Zong *et al*. reported miR-29b having a regulatory influence *via* the PI3K signalling pathway in a GDM rat model [29]. Although this research was not performed in adipose tissue but in liver and in a rodent model, it highlights the interest for identifying non-coding RNA transcripts within this locus in order to understand the transcriptome differences between adipose tissue types.

A limitation of our study, which should be mentioned, is that the analyses were performed in whole tissue samples, as in other comparable studies [11,12,14,24,30–32], and due to the initially limited sample material available, protein expression analysis could not be performed here.

We cannot finally exclude that the reduced gene expression of *PI3KR1* in VAT of GDM patients may be related to differences in the body fat mass and/or distribution or could just be an epiphenomenon, rather than a pathogenic factor. However, our finding is in line with the former observation of *IR* being specifically altered in VAT. Therefore, we suggest a specific role of VAT insulin resistance in the pathophysiology of GDM which should be further explored. In summary and conclusion, VAT reduction of *PI3KR1* expression appears as specific pathogenic co-factor in GDM, pointing to the particular role visceral obesity may play in GDM pathophysiology. However, this is not explainable by altered DNA promoter methylation of *PI3KR1*, characterized here for the first time, in general.

## Abbreviations

BMI: Body-mass-index
CS: Caesarean section
GDM: gestational diabetes mellitus
HOMA-IR: Homoeostatic model assessment of insulin resistance
*IRS1*: insulin receptor substrate 1
*IRS2*: insulin receptor substrate 2
NGT: Normal glucose tolerance
oGTT: Oral glucose tolerance test
*PI3KCA*: phosphoinositide-3-kinase alpha
*PI3KR1*: phosphoinositide-3-kinase subunit p85
*PPIA*: peptidylprolyl isomerase A
SAT: Subcutaneous adipose tissue
VAT: Visceral adipose tissue
